# Editorial: Current Trends in Image Processing and Pattern Recognition

**DOI:** 10.3389/frobt.2021.785075

**Published:** 2021-12-09

**Authors:** KC Santosh

**Affiliations:** PAMI Research Lab, Computer Science, University of South Dakota, Vermillion, SD, United States

**Keywords:** artificial intelligence, computer vision, machine learning, image processing, signal processing, pattern recocgnition

Technological advancements in computing multiple opportunities in a wide variety of fields that range from document analysis (Santosh, 2018), biomedical and healthcare informatics (Santosh et al., 2019; Santosh et al., 2021; Santosh and Gaur, 2021; Santosh and Joshi, 2021), and biometrics to intelligent language processing. These applications primarily leverage AI tools and/or techniques, where topics such as image processing, signal and pattern recognition, machine learning and computer vision are considered.

With this theme, we opened a call for papers on Current Trends in Image Processing & Pattern Recognition that exactly followed third International Conference on Recent Trends in Image Processing & Pattern Recognition (RTIP2R), 2020 (URL: http://rtip2r-conference.org). Our call was not limited to RTIP2R 2020, it was open to all. Altogether, 12 papers were submitted and seven of them were accepted for publication.

In Deshpande et al., authors addressed the use of global fingerprint features (e.g., ridge flow, frequency, and other interest/key points) for matching. With Convolution Neural Network (CNN) matching model, which they called “Combination of Nearest-Neighbor Arrangement Indexing (CNNAI),” on datasets: FVC2004 and NIST SD27, their highest rank-I identification rate of 84.5% was achieved. Authors claimed that their results can be compared with the state-of-the-art algorithms and their approach was robust to rotation and scale. Similarly, in Deshpande et al., using the exact same datasets, exact same set of authors addressed the importance of minutiae extraction and matching by taking into low quality latent fingerprint images. Their minutiae extraction technique showed remarkable improvement in their results. As claimed by the authors, their results were comparable to state-of-the-art systems.

In Gornale et al., authors extracted distinguishing features that were geometrically distorted or transformed by taking Hu’s Invariant Moments into account. With this, authors focused on early detection and gradation of Knee Osteoarthritis, and they claimed that their results were validated by ortho surgeons and rheumatologists.

In Tamilmathi and Chithra, authors introduced a new deep learned quantization-based coding for 3D airborne LiDAR point cloud image. In their experimental results, authors showed that their model compressed an image into constant 16-bits of data and decompressed with approximately 160 dB of PSNR value, 174.46 s execution time with 0.6 s execution speed per instruction. Authors claimed that their method can be compared with previous algorithms/techniques in case we consider the following factors: space and time.

In Tamilmathi and Chithra, authors carefully inspected possible signs of plant leaf diseases. They employed the concept of feature learning and observed the correlation and/or similarity between symptoms that are related to diseases, so their disease identification is possible.

In Das Chagas Silva Araujo et al., authors proposed a benchmark environment to compare multiple algorithms when one needs to deal with depth reconstruction from two-event based sensors. In their evaluation, a stereo matching algorithm was implemented, and multiple experiments were done with multiple camera settings as well as parameters. Authors claimed that this work could be considered as a benchmark when we consider robust evaluation of the multitude of new techniques under the scope of event-based stereo vision.

In Steffen et al.; Gornale et al., authors employed handwritten signature to better understand the behavioral biometric trait for document authentication/verification, such letters, contracts, and wills. They used handcrafter features such as LBP and HOG to extract features from 4,790 signatures so shallow learning can efficiently be applied. Using k-NN, decision tree and support vector machine classifiers, they reported promising performance.

## References

[B1] Santosh KC Antani S. Guru D. S. Dey N. (2019). Medical Imaging Artificial Intelligence, Image Recognition, and Machine Learning Techniques. United States: CRC Press. ISBN: 9780429029417. 10.1201/9780429029417

[B2] Santosh KC Das N. Ghosh S. (2021). Deep Learning Models for Medical Imaging, Primers in Biomedical Imaging Devices and Systems. United States: Elsevier. eBook ISBN: 9780128236505.

[B3] Santosh KC (2018). Document Image Analysis - Current Trends and Challenges in Graphics Recognition. United States: Springer. ISBN 978-981-13-2338-6. 10.1007/978-981-13-2339-3

[B4] Santosh KC Gaur L. (2021). Artificial Intelligence and Machine Learning in Public Healthcare: Opportunities and Societal Impact. Spain: SpringerBriefs in Computational Intelligence Series. ISBN: 978-981-16-6768-8. 10.1007/978-981-16-6768-8

[B5] Santosh KC Joshi A. (2021). COVID-19: Prediction, Decision-Making, and its Impacts, Book Series in Lecture Notes on Data Engineering and Communications Technologies. United States: Springer Nature. ISBN: 978-981-15-9682-7. 10.1007/978-981-15-9682-7

